# Educational Needs of European Intensive Care Nurses with Respect to Multicultural Care: A Mix-Method Study

**DOI:** 10.3390/ijerph19020724

**Published:** 2022-01-10

**Authors:** Aleksandra Gutysz-Wojnicka, Dorota Ozga, Eva Barkestad, Julie Benbenishty, Bronagh Blackwood, Kristijan Breznik, Bojana Filej, Darja Jarošová, Boris Miha Kaučič, Ivana Nytra, Barbara Smrke, Renáta Zeleníková, Beata Dobrowolska

**Affiliations:** 1 Department of Nursing, Faculty of Health Sciences, Collegium Medicum, University of Warmia and Mazury in Olsztyn, Żołnierska 14c Street, 10-561 Olsztyn, Poland; olagut@go2.pl; 2 Department of Emergency Medicine, Faculty of Medicine, The University of Rzeszów, Pigonia 6 Street, 35-310 Rzeszów, Poland; gdozga@poczta.fm; 3 Department of Anaesthesia and Intensive Care Danderyd Hospital, 18882 Stockholm, Sweden; barkestad@gmail.com; 4 Faculty of Nursing, School of Medicine, Hadassah Hebrew University, Jerusalem 91120, Israel; julie@hadassah.org.il; 5 Wellcome-Wolfson Institute for Experimental Medicine, Queen’s University Belfast, 97 Lisburn Road, Belfast BT9 7BL, UK; b.blackwood@qub.ac.uk; 6 International School for Social and Business Studies, Mariborska cesta 7, 3000 Celje, Slovenia; kristijan.breznik@mfdps.si; 7 Research Institute, College of Nursing in Celje, Mariborska cesta 7, 3000 Celje, Slovenia; bojana.filej@gmail.com (B.F.); miha.kaucic@vzsce.si (B.M.K.); barbarasmrke79@gmail.com (B.S.); 8 Department of Nursing and Midwifery, Faculty of Medicine, University of Ostrava, Syllabova 19, 708 00 Ostrava, Czech Republic; darja.jarosova@osu.cz (D.J.); renata.zelenikova@osu.cz (R.Z.); 9 Department of Intensive Medicine and Forensic Studies, Faculty of Medicine, University of Ostrava, Syllabova 19, 708 00 Ostrava, Czech Republic; Ivana.nytra@osu.cz; 10 Department of Holistic Care and Management in Nursing, Faculty of Health Sciences, Medical University of Lublin, Staszica Str. 4–6, 20-081 Lublin, Poland

**Keywords:** ICU, critical care nurses, multicultural care, educational needs, Europe

## Abstract

The aim of the study is the analysis of educational needs of European intensive care nurses (ICNs) with regard to multicultural care. A mixed-method multinational study was performed among 591 ICNs coming from 15 European countries. An online survey was utilised with three research tools: participants’ sociodemographic details, Healthcare Provider Cultural Competence Instrument, and a tool to assess the educational needs of ICU nurses with respect to multicultural care. The highest mean values in self-assessment of preparation of ICU nurses to provide multicultural nursing care and their educational needs in this regard were detected in the case of nurses coming from Southern Europe (M = 4.09; SD = 0.43). With higher age, nurses recorded higher educational needs in the scope of multicultural care (*r* = 0.138; *p* = 0.001). In addition, speaking other languages significantly correlated with higher educational needs related to care of patients coming from different cultures (Z = −4.346; *p* < 0.001) as well as previous education on multicultural nursing care (Z = −2.530; *p* = 0.011). Experiences of difficult situations when caring for culturally diverse patients in ICU were classified into categories: ‘treatment procedures and general nursing care’, ‘family visiting’, ‘gender issues’, ‘communication challenges’, and ‘consequences of difficult experiences’. The educational needs of intensive care nurses in caring for culturally diverse patients are closely related to experiencing difficult situations when working with such patients and their families.

## 1. Introduction

The World Federation of Critical Care Nurses (WFCCN) in the *Brisbane Declaration on Culturally Sensitive Critical Care Nursing* (2016) has defined general principles and recommendations for the care of culturally diverse patients in intensive care units (ICU) [1]. The declaration states that patients from diverse cultures have specific needs and must be cared for by nurses with specialist knowledge, skills, and attitudes [1]. Cultural competences are an important component of the professionalism that nurses must acquire in order to provide the best possible healthcare without disturbing the culturally sensitive areas of the life of the patient and his or her family [2].

Cultural competences are seen as an essential element in providing adequate, effective, and culturally appropriate healthcare; reducing health and care gaps between racial and ethnic groups; and improving healthcare quality, patient satisfaction and treatment outcomes [3]. Satisfying the health needs of a culturally diverse community is also a challenge for leaders of healthcare organisations [4].

At the same time, the term cultural competence is not unequivocally defined by researchers [5], which makes it difficult to choose the optimal methods of educating nurses, planning and organising nursing care, and assessing cultural competences. The majority of the cultural competence models described in the literature contain constructs involving affective, cognitive, and practical domains [3]. In 2002, Burchum [6], using Rodgers’ evolutionary concept analysis method, identified a total of six attributes of cultural competence that are defined in the literature most consistently. These were cultural awareness, cultural knowledge, cultural skill, cultural sensitivity, cultural interaction, and cultural understanding. Additionally, Szen (2014) established that the four attributes of cultural competence, such as sensitivity, awareness, knowledge, and skills, constitute the domains or subscales among 13 of the 15 cultural competence models or assessment instruments described in the literature. These attributes are key elements in the definition of the term cultural competence [3] as proposed by researchers. Furthermore, cultural competence has been consistently recognised by most researchers as a continuous, developmental, evolutionary, evolving, and dynamic process [3]. An integral element of cultural competence is cultural assessment. According to Leininger (2002), to be culturally competent “means to be able to assess and understand culture, care, and health factor and use this knowledge in creative ways with people of diverse or similar lifeways” [7].

The cultural competences of European intensive care nurses (ICNs) were assessed by Dobrowolska et al. [8] with the use of the Healthcare Provider Cultural Competence Instrument (HPCCI) [9]. The authors found that ICNs tended to have high scores in “cultural awareness and sensitivity” but not well-developed cultural skills (behaviour, practice orientation, and patient-centred communication). Similar results were obtained by de Beer and Chipps [10]. These results show in what direction multicultural education for nurses should be tailored.

At the same time, intensive care nurses’ experiences of caring for culturally diverse clients and their families are reported as complex and challenging. In 2008, Høye and Severinsson reported that experiences of Norwegian ICNs when caring for culturally diverse patients are described by the theme ‘cultural diversity and workplace stressors’. This theme included the following categories: ‘impact on work patterns’, ‘communication challenges’, ‘responses to crises and professional status’, and ‘gender issues’ [11]. Then, in 2010, the same authors described conflicts which ICNs experience between professional nursing practice and diverse family cultural traditions [12]. Furthermore, Listerfelt et al. (2019) reported that caring for the relatives of culturally diverse critically ill patients was a challenging experience for ICNs in Sweden due to linguistic and cultural barriers [13]. Intensive care nurses’ experiences in caring for Muslim patients in Saudi Arabia was analysed by Halligan (2006) [14]. The author reported that ICNs who participated in interviews felt that caring for patients of Islamic background posed a professional and personal challenge for them, as they struggled with the stress, frustration, and tensions when caring for culturally diverse patients and their families [14]. Al-Yateem et al. (2015) investigated the experiences of overseas nurses working with Muslim patients in the United Arab Emirates (UAE) and the Kingdom of Saudi Arabia (KSA) [15]. Many of the interviewed nurses reported culture shock, which they experienced during the first six months of work with Muslim patients. In nurses’ opinion, the culture shock was caused by the lack of knowledge about local culture and religion as well as communication barriers resulting from inability to speak Arabic [15].

To our best knowledge, there are no publications describing the educational needs of intensive care nurses related to the care of culturally diverse patients treated in the ICU. The studies carried out so far are aimed at analysing the experiences of intensive care nurses who provided care to culturally diverse, critically ill patients and their families; however, they are usually based on qualitative methodology (e.g., using focus groups or interviews), and they are mostly single-country studies.

The analysis of ICNs’ experiences related to the care of critically ill patients with diverse cultural backgrounds may indicate the educational needs of nurses in the context of providing care that is culturally compatible with the needs of the patient and his or her family. It is therefore advisable to further analyse the experiences of intensive care nurses and their educational needs using qualitative and quantitative research and additionally to collect data from nurses representing culturally diverse world regions.

## 2. Materials and Methods

### 2.1. Aim

The aim of the study is to analyse educational needs of European ICNs with regard to multicultural care.

### 2.2. Study Design and Sample

This is a mixed-method multinational study. Qualitative and quantitative data were collected using an online survey. The convenience sampling method was used. The quantitative part of the study adopted a descriptive, correlational, and cross-sectional design. The qualitative approach utilizing written narrative method was adopted for qualitative data. The study was undertaken as a part of the European project “Multicultural Care in European Intensive Care Units (MICE-ICU)”, funded by the Erasmus plus program (2016-1-PL01-KA202-026615).

The inclusion criteria to participate in the study were (1) nurses working in adult ICUs in European countries, (2) a minimum one year of critical care work experience, and (3) consent to participate in the study. Excluded from the study were anaesthesia nurses, nurses working in accident and emergency units, or those having less than one year of work experience.

The research protocol was approved by the Ethical Committee, University of Rzeszów, Poland (No. 4/4/2017, 6 April 2017). Information about the aim of the study and the process of data collection was provided to respondents in the introduction to the online survey tool. Each participant was assured about the anonymity and confidentiality of the collected data and informed about the right to withdraw from the study at any time. Filling in the online questionnaire was understood as giving consent to participate in the study.

### 2.3. Study Instrument

An online survey was utilised to gather data. The online survey consisted of the three following parts: part 1—participants’ sociodemographic details; part 2—Healthcare Provider Cultural Competence Instrument (HPCCI) to assess the cultural competences of European ICNs; the Cronbach’s alpha for subscales of HPCCI in its original version ranged from 0.72 to 0.92 [9], and in our research, the Cronbach’s alpha for all subscales ranged from 0.57 to 0.90. The findings from this part of the questionnaire were published in 2020 [8]; and part 3—a tool to assess the educational needs of ICU nurses with respect to multicultural ICU care. This part of the survey was developed by a review group appointed by the leader of the MICE-ICU project and consisting of experts from Poland, the Czech Republic, Slovenia, and other European countries associated with EfCCNa.

The tool assessing the educational needs of ICNs was prepared based on the Delphi technique. In accordance with this method, after defining the purpose of the activity, i.e., to develop a tool for assessing the educational needs of ICNs with regard to the care of culturally diverse patients, the leader of the MICE-ICU project prepared an initial set of questions and a scale of answers. A draft version of this tool was prepared based on a review of the literature. The first stage was devoted to locating available research tools that could serve the purpose of assessing the educational needs of intensive care nurses in multicultural care. Electronic databases were queried using EBSCOhost. The following keywords and their combinations were used: educational needs, cultural competence, intensive care nurse, and intensive care unit. An analysis of the review of scientific literature revealed that no research has been published on educational needs of ICNs with regard to cultural diversity. Therefore, at the next stage, a literature review was conducted using keywords, such as adult AND (critical care or intensive care or ICU) AND (nurse or nurses or nursing) AND (experiences or perceptions or attitudes or views) AND cultural diversity. It was assumed that the description of experiences, challenges, and difficulties reported by ICNs in caring for culturally diverse patients/families could serve as a guide to identify the educational needs of intensive care nurses in this area. Based on the results of the literature review [11,12,13,14,15,16], a draft version of the tool assessing educational needs of ICNs was prepared and sent to experts identified by the MICE-ICU project partners. Each partner selected 2 experts in intensive care nursing and education of intensive care nurses. The experts received a draft version of the tool by e-mail and were asked to read it and, in the “Track Changes” mode, add their comments, remarks, and proposals for changes to individual questions and the tool as a whole. After submitting their comments, the experts sent the document to the project leader. All remarks and comments were analysed, and the conclusions were used to prepare the second version of the tool. After two rounds of analysis, the experts reached a consensus as to the number of questions, their content, and the manner of answering them. The final version of the tool assessing educational needs of ICNs with respect to multicultural care included 15 closed-ended questions and one open-ended question aimed at exploring in depth participants’ educational needs, based on analysing difficult situations experienced by nurses when providing care to culturally diverse patients and their families. Respondents answered closed-ended questions on a 5-point Likert scale (0–4), where 0 meant “I absolutely disagree,” 1—“I disagree,” 2—“I neither agree nor disagree” (neutral), 3—“I agree,” and 4—“I absolutely agree.” The higher the score, the stronger educational need. The Cronbach’s alpha for the tool assessing the educational needs of ICNs was 0.91.

The questionnaire was prepared in English and then translated into three national languages (Polish, Slovak, Czech), applying standards of backward and forward translation.

Next, all language versions of the questionnaire were posted for two months on the admin platform dedicated to the MICE-ICU project. The link to the research tool was sent to each of the project partners (Poland, Slovenia, the Czech Republic, and the European Federation of Critical Care Nurses Associations (EfCCNa)) for distribution among potential respondents in each country. Potential respondents received e-mail invitations to participate in the study from their national professional organisations, which acted as MICE-ICU project partners. The invitations were sent to intensive care nurses based on each partner’s database of e-mail addresses.

### 2.4. Data Analysis

The data were analysed using the IBM SPSS Statistics statistical package (v25, IBM, Krakow, Poland). Descriptive statistics was used to describe the participants’ demographic characteristics. The number of observations, mean, median—the most frequent answer and the minimum and maximum values—were used to describe participants’ score regarding the assessment of educational needs. For group comparison, the Mann–Whitney U test (for two groups) or Kruskal–Wallis (for more than two groups) were used. To determine the relationship between variables, a Spearman’s rank-order correlation coefficient was used, and to determine correlations between variables with a normal distribution, the Pearson product-moment correlation coefficient was calculated. The obtained results were considered statistically significant at the *p* < 0.05 level.

The qualitative data were analysed using the approach proposed by Creswell (2013) [17]. This approach involves analysing written narrative from specific to general ones and involving multiple levels of analysis. The data were hand coded by the researcher. First, all answers to the question “As an ICU nurse, I have personally experienced a difficult situation when providing care to culturally diverse patients—give a short description of this difficult situation” were printed, and each answer was marked with the code of the participant’s country (PL-ICN, SL-ICN, Cz-ICN, and EN-ICN, where PL-ICN meant answers from ICU nurses from Poland, SL-ICN from Slovenia, Cz-ICN from the Czech Republic, and EN-ICN from other European countries (southern and northern Europe)). The collected descriptions of difficult situations were then read separately in full by two authors. Notes were made with general comments on the type of topics/problems reported. Re-reading all the situation descriptions and interpreting the content of the individual sentences made it possible to identify new/hidden meanings. After reading all the descriptions twice, identification of meaning units started as well as naming/coding of each of the meaning units identified in the sentences/fragments of the description. After further reading of all descriptions, the identified meaning units were thematically grouped due to common/similar meanings. Groups-of-meaning units served as the basis for the formulation of thematic categories within which subcategories were distinguished. The authors’ analyses were merged, and any discrepancies were discussed and agreed within the group of other authors. The steps taken in this study were as follows:

Step 1. Organise and prepare the data for analysis. Access to written answers to open-ended questions was granted. All respondents’ answers were printed and divided according to the respondent’s country of residence.

Step 2. Read all the data. All descriptions obtained from each country were read in full. A note was made on the researcher’s initial reflections on the categories of the themes/problems.

Step 3. Start coding all of the data. Each answer was reread, the content was analysed, the meanings were interpreted, and the codes were manually assigned to the individual fragments of the answers. Notes and comments were prepared on a regular basis.

Step 4. Use the coding process to generate a description of the people as well as categories or themes for analysis. Each response and the codes assigned were re-analysed, and the codes were combined into groups to identify common categories and sub-categories.

Step 5. Advance how the description and themes will be represented in the qualitative narrative. It was assumed that the identified categories would be presented in the form of a description and in a summary table.

Step 6. The final step in data analysis. Make an interpretation in qualitative research of the findings or results. It was assumed that the identified categories regarding experiences of difficult situations would be used to determine the educational needs of intensive care nurses in the care of culturally diverse patients.

## 3. Results

### 3.1. Participants

A total of 591 nurses participated in the study, including *n* = 155 (26.22%) from Poland, *n* = 221 (37.39%) from the Czech Republic, *n* = 97 (16.41%) from Slovenia, and *n* = 118 (19.96%) from other European countries, including northern, *n* = 90 (15.22%), and southern Europe, *n* = 28 (4.73%). Women constituted the vast majority of the respondents: *n* = 518 (87.64%). The education of the nurses working in the ICU varied, with the majority being Diploma Nurse, *n* = 145 (24.5%); having Bachelor Degree in Nursing, *n* = 123 (20.8%); or Master Degree in Nursing, *n* = 110 (18.6%). The vast majority of the respondents had not previously completed any multicultural nursing course: *n* = 520 (87.99%). The majority of the respondents declared knowledge of foreign languages: *n* = 400 (67.68). Most of the respondents considered themselves religious: *n* = 356 (60.24%). Details are presented in Table 1.

### 3.2. The Quantitative Analysis

#### 3.2.1. Self-Assessment of Knowledge and Skills of ICU Nurses to Care for Patients Who Come from Different Cultures and Their Educational Needs in This Regard

The highest mean values in self-assessment of preparation of ICU nurses to provide appropriate nursing care to culturally diverse patients and their educational needs in this regard was detected in case of nurses coming from Southern Europe (Me = 4.00; SD = 0.43). The difference is statistically significant (<0.001). Details are presented in Table 2.

#### 3.2.2. Educational Needs Regarding Knowledge/Skills of Other Cultures

In the entire group of 591 surveyed ICU nurses, 293 nurses (49.57%, “agree” and “strongly agree”, counted together) declared that they would like to expand their knowledge of other cultures (Table 3).

However, only 163 nurses (27.3%) indicated different ethnic and religious groups that they would like to get to know better. The total number of different ethnic and religious groups reported by all respondents was 347 (respondents could give more than one example). Intensive care nurses from all countries were by far the most likely to report educational needs to increase their knowledge of the sociocultural characteristics of Arab culture (136; 39.19%). Additionally, ICNs from central and eastern European countries expressed the need to broaden their knowledge of Roma and Jehovah’s Witnesses cultures (56, 16.13% and 44, 12.68%, respectively) (Table 4).

#### 3.2.3. Educational Needs of ICU Nurses Regarding Multicultural Nursing Care and Their Sociodemographic Characteristic

Relationships between sociodemographic characteristics and educational needs of ICU nurses are reported in Table 5. Correlations showed that with higher age, nurses recorded higher educational needs in the scope of multicultural care (*r* = 0.138; *p* = 0.001). Additionally, speaking other languages significantly correlated with higher educational needs related to care of patients coming from different cultures (Z = −4.346; *p* < 0.001) as well as previous education on multicultural nursing care (Z = −2.530; *p* = 0.011). Nurses who rarely visited other countries reported lower educational needs (*rho* = −0.186; *p* < 0.001).

#### 3.2.4. Educational Needs Based on the Experience of a Difficult Situation When Caring for Culturally Diverse Patients in ICU

To identify educational needs of ICNs based on their experience in caring for culturally diverse patients in ICU, at first, all participants were asked if they personally experienced any difficult situation when providing care to culturally diverse patients. Over one-third of nurses (*n* = 230; 38.9%) have personally experienced a difficult situation when providing care to culturally diverse patients. Difficult situations were far more frequently confirmed by ICNs from southern, *n* = 21 (75% “agree” and “strongly agree” answers), and northern Europe, *n* = 64 (71.1% “agree” and “strongly agree” answers) (Table 6).

### 3.3. The Qualitative Analysis

Experiences of difficult situations when caring for culturally diverse patients in ICU were classified into five categories describing the causes and effects of these stressful situations. The categories were identified as follows: ‘treatment procedures and general nursing care’, ‘family visiting’, ‘gender issues’, ‘communication challenges’, and ‘consequences of difficult experiences’ (Table 7).

The ‘treatment procedures and general nursing care’ category describes difficult situations related to the nurse’s insufficient knowledge about culturally acceptable diagnostic and therapeutic procedures and the standards and methods of providing culturally sensitive nursing care in different cultures and ethnic groups. This category includes subcategories, such as ‘death and dying practices’, ‘blood transfusion and transplantation’, ‘end-of-life care’, ‘hygiene’, ‘dietary habits’, and ‘praying’. Examples of difficult situations reported by the respondents include the following:

*“Lack of knowledge about the Hindu final farewell customs resulted in complete chaos in the ward when about 60 people arrived for the final farewell”* (PL-ICN).

*“Taking care of a child* [of Jehovah’s Witnesses parents] *who was seriously ill and needed a lot of blood transfusions, surgeries and all kinds of treatments. All of this was done with the parents disagreeing with everything we did. They were told that if they didn’t agree they might lose custody of the child because the child wouldn’t survive without the treatments. It was very hard and all communication with the patients made this case very difficult”* (EN-ICN).

The ‘family visiting’ category includes a description of stressful situations related to the different and difficult expectations of families of culturally diverse patients. This category includes issues related to differences in ‘family structure’ and ‘family obligations towards a sick family member’. Sample descriptions of difficult situations include the following:

*“Refusal by a Roma family to leave the patient’s room during a medical procedure”* (PL-ICN).

*“Also, I haven’t always understood differences between family sizes. For example, a Finnish family is usually a small group but Asian families can be quite large”* (EN-ICN).

The ‘gender issues’ category describes the difficult experiences of nurses related to the varied perception of the position of women and men in different religions and cultures. In this category, the following subcategories have been distinguished: ‘gender-based expectations of care’ and ‘men as decision makers’. Examples of such difficult situations are as follows:

*“Patient’s wife wanted a male nurse to treat her husband in every shift; we couldn’t provide enough male nurses”* (EN-ICN).

*“In the area of gynaecological-obstetric care, I observed husband’s strong objections when a male doctor was present in the room”* (Cz-ICN).

In the communication challenges category, two subcategories were reported: communication barriers and ensuring effective communication and providing information are distinguished. Examples of difficult situations include the following:

*“Inability to effectively communicate with the patient’s family, concerns about health and life, and the absence of an interpreter to aid in communication between the staff and the family as well as the patient himself. There were patients from Ukraine, the United Arab Emirates, Vietnam, Mongolia or Bulgaria, who also communicated in their native language only”* (Cz-ICN).

The ‘consequences of difficult experiences’ category describes the impact of difficult situations related to the care of culturally diverse patients on the personal and professional functioning of nurses and the reactions of patients and their families. This category includes subcategories, such as ‘professional dilemmas’, ‘risk of disrespectful care’, ‘work-related stress’, and ‘culturally based conflict’ arising from patients’ and their families’ distrust towards staff, non-compliance with ICU instructions and regime, refusal to consent to selected procedures, and causing damage to hospital property and other patients. Examples of descriptions are as follows:

*“An Arab patient who required constant care and his behaviour towards nurses and doctors was aggressive”* (Cz-ICN).

*“So far we haven’t had much contact with other ethnic groups except Roma whose certain preferences are for us, “average Slovenians”, (almost) unacceptable. It is difficult to impose rules on ‘when specifically visits are allowed, how many people can enter the ward, how they must behave towards staff,’ etc. They should therefore be instructed in this respect and made aware of the rights and obligations applicable in hospitals”* (SL-ICN).

Qualitative analysis of the collected meaning units in the context of the educational needs of intensive care nurses (ICNs) allowed for the identification of their educational needs in terms of knowledge, skills, and competences (Table 8). According to the European Qualifications Framework [18], it was assumed that knowledge refers to the outcome of the assimilation of information through learning. Knowledge is the body of facts, principles, theories, and practices that is related to a field of work or study. Skill refers to the ability to apply knowledge and use know-how to complete tasks and solve problems. In the context of the European Qualifications Framework, skills are described as cognitive or practical. Competence refers to the proven ability to use knowledge, skills, and personal, social, and/or methodological abilities in work or study situations and in professional and personal development [18].

## 4. Discussion

The aim of this study was the analysis of educational needs of European ICNs related to multicultural care, using quantitative and qualitative methods. Considering the level of educational needs of ICNs in our study, it can be assumed that they are interested in broadening their knowledge and skills regarding care for patients/their family coming from diverse cultures. Qualitative analysis of difficult situations experienced by intensive care nurses and the educational needs resulting from these experiences confirmed the results of quantitative data. The use of qualitative methods allowed the identification of educational needs with respect to knowledge regarding communication style and providing effective information/communication, death and dying, blood transfusion and transplantation, family structure and decision-making rules in different cultures, culture- and religion-related rules, and special cultural needs of culturally diverse patients/family, end-of-life care, dietary practices, and praying. In the field of skills, the identified educational needs were related to the following abilities: organise culturally acceptable care within limited resources, coping with gender issues, and solving problems, managing culturally based conflicts, and unfamiliar situations. In terms of competences, educational needs were identified related to the lack of mastery of effective strategies for dealing with ethical dilemmas and stress.

The experiences of intensive care nurses in caring for critically ill patients in the ICU have also been analysed by other authors [13,14,15] and are in line with results achieved in our qualitative analysis regarding the type of identified difficult situations experienced by intensive care nurses caring for culturally diverse patients. For example, Listerfelt et al. (2019), during focus group interviews, identified one main theme which described ICNs’ experiences: ‘facing the unfamiliar’. This theme constituted a set of linguistic and cultural challenges that were further classified into categories such as the following: ‘relatives taking up space’, ‘communication as a challenge’, ‘crisis reactions cause drama, and providing equal’ and ‘personal adjusted care’ [13]. Generally speaking, the most challenging experience for ICNs are related to the role of the family of the client in the process of health care, importance of religion and religion-related traditions and practices, and communication with the patients and their families.

Nurses from the northern and southern European countries reported higher need in education regarding care for patients/their family of different cultural roots compared to nurses representing the rest of European countries. This may indicate a higher level of cultural awareness and sensitivity of these nurses due to the significantly higher frequency of experiencing difficult situations by ICNs from northern and southern Europe as compared to nurses from the Czech Republic, Poland, and Slovenia. This is in line with the results of the study by Kim (2013) [19], who reported that factors that influence nurses transcultural self-efficacy are the degree of interest in multi-culture, educational needs for cultural competence in nursing, and the degree of experience in caring for multi-cultural clients. In a study of the cultural competence of intensive care nurses (ICNs) from central, northern, and southern Europe conducted by Dobrowolska et al. (2020) [9], the authors found that ICNs residing in southern Europe recorded higher scores than nurses from other European regions, with the differences between regional groups being statistically significant. There was also a statistically significant difference in scores on the self-assessment subscale, which involves reflecting on one’s own practice. Importantly, it is believed that reflection on provider’s beliefs, values, and attitudes can further the provider’s cultural competence and active engagement in inclusive practices. Similar conclusions were also formulated by de Beer and Chipps (2014), who assessed the cultural competences of ICNs in South Africa. The authors used the Inventory to Access the Process of Cultural Competence—Revised (IAPCC-R) and concluded that the majority of ICNs were culturally aware but were not yet competent. However, in the summary, it was stated that cultural awareness was the first step in the process of developing cultural competence [10].

When providing multicultural education for nursing students, to achieve the learning outcomes and develop cultural competences, education organisers and teachers should focus on innovative teaching strategies and methods. Oikarainen et al. conducted a systematic literature review to identify the best evidence on the types of educational interventions to improve nurses’ cultural competence and on the effectiveness of these interventions. The authors reported that currently, it is not possible to identify the best methods, forms of teaching, educational content, and educational interventions to develop nurses’ cultural competence [20]. However, when reflecting on the results reported in our study, considering positive correlation between previous multicultural nursing care course and educational needs of ICNs in the scope of providing multicultural care as well as negative correlation between frequency of visiting other countries and educational needs regarding multicultural nursing care, this implies clear suggestions for organisations of nursing education, who should promote international practical training possibilities as a source of multicultural nursing care knowledge, skills, and competence. Results of other studies underlined the role of skilled teachers in multicultural nursing education [21] and importance of uncomplicated education resources to equip nurses with skills to address problematic cultural situations [22]. Oikarainen et al. [20] highlighted that future studies should focus on reporting specific components of interventions that result in an increase in cultural competence. What is important and has been underlined by Camphina-Bacote in her model for cultural competence is that developing cultural competence it is not an event but the continuous process [23].

### Limitations

There are some limitations of our study. The first one is the purposeful sampling methodology and method of data collection that we used. The invitation to participate in this study was sent to national representatives of critical care nursing associations that are European members of EfCCNa. Because we did not receive responses from all countries, results may not reflect the educational needs of all European intensive care nurses. The second one is the voluntary participation through online surveying. Since the study is not based on a random sample, there is a risk of participation bias. Nurses who are not comfortable with the Internet usage may be underrepresented and may have different viewpoints. The third limitation that should be mentioned is the lack of the whole process of validation of the instrument measuring educational needs prior to the research. The Cronbach’s alpha was calculated for this tool; however, other psychometric properties.

## 5. Conclusions

The educational needs of intensive care nurses in caring for culturally diverse patients are closely related to experiencing difficult situations when working with such patients and their families. The more often the nurses experienced difficult situations, the more often they expressed a need to deepen and develop their knowledge and skills.

The identified educational needs have been confirmed in both qualitative and quantitative research. The educational needs of intensive care nurses vary according to the region/country they come from. What is important is that the more nurses know about the topic of care for patients who come from different cultures, the more they want to develop their expertise regarding multicultural nursing care. This trend was clearly visible in the case of nurses from southern and northern Europe.

These findings imply an important message for multicultural nursing education. Nursing students should be more often exposed during their clinical education to experience of nursing care on culturally diverse patients/families in order to “feel” the need for constant development of their cultural competence not only during their formal professional education but also beyond it.

## Figures and Tables

**Table 1:** Sociodemographic characteristics of the surveyed nurses (*n* = 591).

Variables	*n*	%
Gender		
Female	518	87.64
Male	73	12.36
Speaking other languages		
Yes	400	67.68
No	191	32.32
Previous education on multicultural nursing		
Yes	71	12.01
No	520	87.99
Visiting other countries		
Regularly	77	13.03
Very often	80	13.54
Often	180	30.46
Seldom	224	37.90
Never	30	5.07
Education		
Health Assistant	70	11.8
Registered nurse	145	24.5
Bachelor’s degree in nursing	123	20.8
Master’s degree in nursing	110	18.6
Specialisation	125	21.2
PhD	11	1.9
Lack of answer	7	1.2
Religious person		
Yes	356	60.24
No	235	39.76

**Table 2:** Self-assessment of knowledge/skills of ICU nurses regarding multicultural care and their educational needs in this scope.

Educational Needs	Total		Czech Republic		Poland		Slovenia		Northern Europe		Southern Europe/Levant		Statistic	
	Me	SD	Me	SD	Me	SD	Me	SD	Me	SD	Me	SD	H	*p*
	3.80	0.57	3.63	0.53	3.94	0.62	3.81	0.62	3.88	0.44	4.00	0.43	**40.924**	**<0.001**

Legend: Me, median; SD, Standard deviation; H, Kruskal–Wallis test; *p* < 0.05 level was considered statistically significant.

**Table 3:** Educational needs regarding knowledge/skills of other cultures.

As an ICU Nurse, I Should Know More about Sociocultural Characteristics of Different Ethnic and Religious Groups	Czech Republic		Poland		Slovenia		Northern Europe		Southern Europe/ Levant	
	*n*	%	*n*	%	*n*	%	*n*	%	*n*	%
Strongly disagree	2	0.9	1	0.6	2	2.1	0	0.0	0	0.0
Disagree	24	10.9	15	9.7	6	6.2	2	2.2	0	0.0
Neutral	121	54.8	43	27.7	32	33.0	32	35.6	6	21.4
Agree	65	29.4	72	46.5	42	43.3	41	45.6	17	60.7
Strongly agree	9	4.1	16	10.3	12	12.4	14	15.6	5	17.9
N/A	0	0.0	8	5.2	3	3.1	1	1.1	0	0.0
Total	221	100.0	155	100.0	97	100.0	90	100.0	28	100.0

**Table 4:** Ethnic and religious groups indicated by ICU nurses that they would like to get to know better.

Ethnic and Religious Groups	Czech Republic		Poland		Slovenia		Northern/Southern Europe	
	*n*	%	*n*	%	*n*	%	*n*	%
Albanian	0	0.0	0	0.0	2	4.7	0	0.0
American	1	0.7	0	0.0	0	0.0	0	0.0
English	1	0.7	1	1.1	0	0.0	0	0.0
Asian	4	2.8	0	0.0	0	0.0	0	0.0
Buddhist	2	1.4	4	4.3	1	2.3	1	1.4
Chinese	1	0.7	0	0.0	1	2.3	0	0.0
Christian	6	4.2	2	2.2	1	2.3	6	8.6
Ethiopian	0	0.0	0	0.0	0	0.0	5	7.1
Polish	0	0.0	0	0.0	0	0.0	1	1.4
Mormons	0	0.0	0	0.0	1	2.3	0	0.0
Hindu	3	2.1	2	2.2	0	0.0	4	5.7
Arabic	49	34.5	32	34.8	17	39.5	38	54.3
Mongolian	1	0.7	0	0.0	0	0.0	0	0.0
Roma	27	19.0	17	18.5	9	20.9	3	4.3
Orthodox Church	0	0.0	6	6.5	0	0.0	1	1.4
Russian	2	1.4	0	0.0	0	0.0	1	1.4
Jehovah’s Witnesses	17	12.0	15	16.3	10	23.3	2	2.9
Ukrainian	3	2.1	1	1.1	0	0.0	0	0.0
Vietnamese	15	10.6	0	0.0	0	0.0	0	0.0
Jewish	10	7.0	12	13.0	1	2.3	8	11.4
Total *	142	100.0	92	100.0	43	100.0	70	100

* Respondents could give more than one example of different ethnic and religious groups.

**Table 5:** Nurses sociodemographic characteristics and educational needs regarding multicultural nursing care.

	Age		Work Experience in ICU		Speaking Other Languages						Previous Education on Multicultural Nursing						Visits Other Countries *	
					Yes		No		U Mann- Whitney		Yes		No		U Mann- Whitney			
	*r*	*p*	*r*	*p*	Me	SD	Me	SD	Z	*p*	Me	SD	Me	SD	Z	*p*	*rho*	*p*
Educational needs	**0.138**	**0.001**	0.074	0.081	3.81	0.55	3.63	0.58	**−4.346**	**<0.001**	3.88	0.48	3.75	0.58	**−2.530**	**0.011**	**−0.186**	**<0.001**

Legend: *r*, Pearson test; *rho*, Spearman; * 1, regularly; 5, never; Me, median; SD, standard deviation; *p* < 0.05 level was considered statistically significant.

**Table 6:** Experience of a difficult situation when caring for culturally diverse patients in ICU.

As an ICU Nurse, I Have Personally Experienced a Difficult Situation When Providing Care to Culturally Diverse Patients	Czech Republic		Poland		Slovenia		Northern Europe		Southern Europe/Levant	
	*n*	%	*n*	%	*n*	%	*n*	%	*n*	%
Strongly disagree	8	3.6	7	4.5	16	16.5	0	0.0	0	0.0
Disagree	50	22.6	27	17.4	17	17.5	6	6.7	1	3.6
Neutral	88	39.8	33	21.3	27	27.8	18	20.0	6	21.4
Agree	38	17.2	50	32.3	22	22.7	47	52.2	16	57.1
Strongly agree	14	6.3	12	7.7	9	9.3	17	18.9	5	17.9
N/A	23	10.4	26	16.8	6	6.2	2	2.2	0	0.0
Total	221	100.0	155	100.0	97	100.0	90	100.0	28	100.0

**Table 7:** Experiences of a difficult situation when caring for culturally diverse patients.

Theme	Experiences of Difficult Situations When Caring for Culturally Diverse Patients				
Categories	Treatment procedures and general nursing care for culturally diverse patients	Family visiting	Gender issues	Communication challenges	Consequences of experiencing a difficult situation
Subcategories	‑ Death and dying practices ‑ Blood transfusion and transplantation ‑ End-of-life care ‑ Hygiene ‑ Dietary habits ‑ Praying	‑ Family structure ‑ Family obligations towards a sick family member	‑ Gender-based expectations of care ‑ Men as decision makers	‑ Communication barriers ‑ Ensuring effective communication and providing information	‑ Professional dilemma ‑ Risk of disrespectful care ‑ Work-related stress ‑ Professional frustration and dissatisfaction ‑ Culturally based conflict between nurses and patient/family

**Table 8:** Educational needs related to caring for culturally diverse patients in ICU.

Theme	Caring for Culturally Diverse Patients in ICU		
**Categories**	Learning requirements/educational deficiencies related to caring for culturally diverse patients in ICU		
**Subcategories**	Knowledge—principles, theories, and procedures	Skills/cognitive and practical—to apply knowledge and use know-how to complete tasks and solve problems	Competences—abilities to provide culturally sensitive care and to ensure continuous personal and professional self-development
**Code**	‑ Communication-related principles and preferences in different cultures ‑ Death and dying practices in different cultures ‑ Blood transfusion and transplantation in different cultures ‑ Family structure and decision-making rules in families from different cultures ‑ Culture/religion-related principles of providing nursing care ‑ End-of-life care preferences in different cultures ‑ Dietary practices in different cultures	‑ Organise culturally acceptable care within limited resources ‑ Effectively communicate and provide meaningful information (with/without interpreter) ‑ Copy with families expectations ‑ Copy with gender issues ‑ Manage culture-based conflicts and unfamiliar situations	‑ Professional dilemmas ‑ Professional frustration and dissatisfaction ‑ Work-related stress ‑ Increased workload ‑ Recognizing limitations and culturally based stereotypes

## Data Availability

The data presented in this study are available on request from the corresponding author.
